# Quantum deep learning by sampling neural nets with a quantum annealer

**DOI:** 10.1038/s41598-023-30910-7

**Published:** 2023-03-09

**Authors:** Catherine F. Higham, Adrian Bedford

**Affiliations:** 1 School of Computing Science, University of Glasgow, Glasgow, G12 8QQ UK; 2 OxBrdgRbtx Ltd, Stratford-upon-Avon, CV37 6XU UK

**Keywords:** Mathematics and computing, Computational science

## Abstract

We demonstrate the feasibility of framing a classically learned deep neural network as an energy based model that can be processed on a one-step quantum annealer in order to exploit fast sampling times. We propose approaches to overcome two hurdles for high resolution image classification on a quantum processing unit (QPU): the required number and the binary nature of the model states. With this novel method we successfully transfer a pretrained convolutional neural network to the QPU. By taking advantage of the strengths of quantum annealing, we show the potential for classification speedup of at least one order of magnitude.

Deep learning approaches are being applied and refined for classification tasks across many data types (images, video and audio) with high levels of success^1–3^. There are many applications where classically trained convolutional neural networks (CNNs) perform effectively, sometimes better than human experts^4–6^. However in some circumstances, including security, defence and automated transport, safety-critical benefits would arise if classifications can be computed more quickly and classification models checked more thoroughly. We give a proof of principle demonstration that quantum annealing (QA) has the potential to address this issue.

## Background and related work

### Quantum annealing technology

The current D-Wave Advantage System 4.1 contains around 5,000 qubits and 35,000 couplers^7^. These qubits can be physically coupled to form networks with real-valued coefficients denoting coupling strength and individual on/off biases. Connectivity per qubit is limited to 15 couplers and a Pegasus graph architecture but can be extended by forming chains of qubits. These coefficients define and constrain relationships between qubits and form a quadratic binary model capable of expressing a range of behaviours. The parameter coefficients of a given model are embedded on the D-Wave network and by posing the problem as energy minimisation, quantum annealing is used to find the low energy states (on/off) of the qubits and hence the most likely formations. The ability to rapidly sample from many states and hence characterise the shape of the energy landscape is a key benefit of this technology.

### Underlying quantum physics: the Hamiltonian

A classical Hamiltonian gives a mathematical description of some physical system in terms of its energies. For most non-convex Hamiltonians, finding the minimum energy state is an NP-hard problem that classical computers cannot solve efficiently. In quantum annealing, the system begins in the lowest-energy state of an initial Hamiltonian, *A*, and as it anneals introduces the problem Hamiltonian, *B*. To do this, a time-like parameter *s* and annealing functions *A*(*s*) and *B*(*s*) are introduced such that $A(0) \gg B(0)$ and $B(1) \gg A(1)$. Hence, as the system is annealed, *A*(*s*) decreases, *B*(*s*) increases and we approach the desired solution states. This approach has the potential for significant benefits in terms of both speed and accuracy, compared with classical computing technology.

For the D-Wave system^7^, the Hamiltonian is expressed as follows

1 $$\begin{aligned} H = \frac{A(s)}{2} \left( \sum _i {\hat{\sigma }}^{(i)}_x \right) + \frac{B(s)}{2} \left( \sum _i h_i {\hat{\sigma }}^{(i)}_z + \sum _{i<j} J_{i,j} {\hat{\sigma }}^{(i)}_z {\hat{\sigma }}^{(j)}_z \right) , \end{aligned}$$

where ${\hat{\sigma }}^{(i)}_{x,z}$ are Pauli matrices operating on a qubit $q_i$, and $h_i$ and $J_{i,j}$ are the qubit local field values or biases and coupling strengths or weights, respectively. In the final state, the qubits take values of either 0 or 1. Hence this provides a classical solution for the problem Hamiltonian *B* defined by the biases, $h_i$, and the coupling weights, $J_{i,j}$.

### Machine learning models: Boltzmann machines

Boltzmann machines^8^ are probabilistic models with an energy-based distribution that define a probability for each of the *N* discrete states in a binary vector given by

2 $$\begin{aligned} p(x) = \frac{1}{Z} \exp (-E(x;\theta )). \end{aligned}$$

 

  Here $E(x;\theta )$ is an energy function parameterised by $\theta$, and $Z =\sum _x \exp (-E(x;\theta ))$ is the normalizing coefficient, also known as the partition function, which ensures that *p*(*x*) sums to 1 over all the possible states of *x*. The energy function can be represented via a quadratic form $x^TQx$ in which the upper-triangular matrix $Q=Q_{\theta }$ encapsulates the parameters of the quadratic energy function defined by

3 $$\begin{aligned} E(x;\theta ) = x^T Q x = \sum _i^N q_{i,i} x_i + \sum _{i < j}^N q_{i,j} x_i x_j, \end{aligned}$$

where $q_{i,i}$ and $q_{i,j}$ are the biases and correlation weights respectively. This expression makes clear the connection between Boltzmann machines, quadratic binary models, and the final Hamiltonian in the second part of Eq. (1). This suggests that Boltzmann machines are good candidates for training and evaluation on the D-Wave quantum annealer as the problem can be framed in such a way that the solution to the final Hamiltonian is the low energy state of the problem. In this work we are interested in transferring classically learned weights to a quantum system for speed up and hence we focus on the evaluation task.

#### Restricted Boltzmann machines

A restricted Boltzmann machine (RBM) is a special type of Boltzmann machine with a symmetrical bipartite structure^9^. The set of binary variables is divided into visible (input), *v*, and hidden, *h*, variables. We will show, we believe in the context of quantum annealing for the first time, that activation of the hidden nodes within a RBM is equivalent to activation of the hidden nodes by a sigmoid layer, with fully connected or convolutional filters, within a neural network. The hidden variables allow for more complex dependencies among visible variables and are used to learn a stochastic generative model over a set of inputs. All visible variables connect to all hidden variables, but no variables in the same layer are linked. In the classical setting this limited connectivity makes inference and therefore learning easier because analytical expressions can be found for the conditional probabilities.

### Related work

In their survey paper, Nath et al.^10^ review the application domains where a physical quantum annealer has been used to train machine learning classifiers and discuss the advantages and problems of quantum over classical. The image recognition applications include training with RBMs. In another application area, remote sensing imagery, Boyda et al.^11^ train a classifier based on boosting, the tactic of building a strong classifier as an optimally weighted combination of weak classifiers. This classifier is an optimal voting subset of weak classifiers and is adapted for the QPU. Several authors have explored the connection between RBMs and quantum annealers. Adachi and Henderson^12^ investigated estimating the model expectations of RBMs using samples on a 512-qubit D-Wave machine and successfully trained a model with up to 32 visible nodes and 32 hidden nodes per RBM layer. In their tests, they found that this approach achieves comparable or better accuracy with significantly fewer iterations of generative training than conventional contrastive divergence based training^13^ on a coarse-grained version of the MNIST data set^14^. Sleeman et al.^15^ also investigated the feasibility of using the D-Wave as a sampler for machine learning. Their work described a hybrid system that combined a classical deep neural network autoencoder with a quantum annealing RBM. Their method overcame two key limitations in the 2000-qubit D-Wave processor, namely the limited number of qubits available to accommodate typical problem sizes for fully connected quantum objective functions and the restriction to samples that are binary pixel representations. Their hybrid autoencoder approach indicated advantage for quantum annealing relative to the use of a classical computer implementation for image-based machine learning and hinted at even more promising results for the next generation D-Wave quantum system.

In^16^, the model expectation of gradient learning for RBM was calculated using a quantum annealer (D-Wave 2000Q), giving much faster results than Markov chain Monte Carlo used in contrastive divergence. Most Boltzmann machines use restricted topologies that exclude looping connectivity, to avoid complex distributions that are difficult to sample. The work in^17^ used an open-system quantum annealer to sample from complex distributions and implemented Boltzmann machines with looping connectivity.

Caldeira et al. compress galaxy images using principal component analysis to obtain 64-bit images and develop and train an RBM classifier to distinguish between two classes (spiral/rounded smooth galaxies). They compare their results using quantum annealing to a range of methods (MCMC, gibbs sampling, simulated annealing and gradient boosted trees). They impose a regulated quadratic training objective to select an optimal voting subset. The votes of the subset define the classification.

In this work our starting point is a classically trained CNN that maps real valued features to a classification label. Unlike^12,15,16,18^ we do not train on the QPU but investigate transferring the CNN problem to the QPU in order to obtain solutions. Hereby exploiting the strengths of the QPU in the classification task. However we encounter similar issues: qubit number, binary nature and connectivity. Our work looks at how these challenges can be addressed on the recent D-Wave Advantage in the context of deep learning transfer with a view to classification speedup.

### Contributions

The main contribution of this work is to show that a trained artificial neural network can be transferred to a quantum computing setting, giving a potential increase in classification speed. To do this we use the framework of energy minimisation involving an appropriate quadratic form, where quantum annealing provides samples from the low energy, most likely model states. Results, obtainable in microseconds rather than milliseconds, can be used to estimate a predictive class score. We show how to design a quadratic binary model, the engine of quantum annealing, to behave like a neural network, combining deep learned parameters and layer structures from a classically trained neural network with quadratic binary model parameters. Constraint parameters are adapted so that designated class units act together as a softmax classification unit. We then test this approach on digit image data by finding an appropriate embedding on the D-Wave QPU and transferring the coupling and bias parameters from the classical model to the qubits. Key barriers to scaling up to high resolution image/video processing are the binary nature of the variables, the number of qubits that could be assigned to visible units and the limited number of couplers per qubit. We address these issues by showing how real-valued features can be introduced into the system. Another novel aspect of our work is that we revisit RBMs and use them to extract binary features from real valued data so that the images can be processed on the QPU. Others have used RBMs in this way for training. We make use of classical algorithms (contrastive divergence and backpropagation) to increase the size of the features that can be loaded on to the QPU and to achieve classification with 10 classes.

## Methods: framing classification as quadratic unconstrained binary optimisation (QUBO)

A classification problem with *K* classes can be addressed using a neural network composed of sequential functions to map each data point $x \in {\mathbb {R}}^N$ to *K* real valued numbers. We focus on a neural network classifier, $f_{\theta }(x)$, that is defined using two mapping functions: feature extraction,

4 $$\begin{aligned} f^1:x \rightarrow \sigma (Wx + b), \end{aligned}$$

where *W* is a weight matrix and *b* is a bias vector and $\sigma (x)$ is a nonlinear activation function, and classification,

5 $$\begin{aligned} f^2:x \rightarrow W^c x + b^c, \end{aligned}$$

where $W^c$ is a weight matrix and $b^c$ is a bias vector. The neural network classifier can then be written

6 $$\begin{aligned} f_\theta (x) = f^2(f^1(x)). \end{aligned}$$

   

The parameters $\theta = \{W,b,W^c,b^c\}$ are optimised through training and $f_\theta (x)$ is used as part of a softmax activation to determine a class probability score:

7 $$\begin{aligned} p_\theta (class=i|x) = \frac{\exp (f_\theta (x)_i)}{\sum _j \exp (f_\theta (x)_j) }. \end{aligned}$$

    

The form of the nonlinear activation function, $\sigma$, can be chosen to suit the problem. We choose to work with a sigmoid activation layer

8 $$\begin{aligned} \sigma (x)= 1/(1+\exp (-x)), \end{aligned}$$

as this type of activation can be reproduced on the QPU, as shown below.

Adopting the notation where *v* denotes *N* input or visible binary nodes and *h* denotes *M* feature nodes or hidden nodes, and for $W \in {\mathbb {R}}^{M \times N}$ and $b \in {\mathbb {R}}^{M}$, the nonlinear map, $f^1(v)$, maps $v \in {\mathbb {B}}^{M}$ to a feature $h \in {\mathbb {R}}^{M}$. Similarly for $W^c \in {\mathbb {R}}^{K\times M}$ and $b^c \in {\mathbb {R}}^{K}$, $f^2(h)$, maps *h* to a class feature $h^c \in {\mathbb {R}}^{M}$. The class feature is turned into a score following Eq. (7). To transfer the feature extraction and classification task to the QPU the network nodes are each assigned to a qubit, the programmable qubit local field and between qubit coupling values are set and the network is embedded on the quantum machine.

The quantum annealer will be used in two different scenarios. First, we will consider feature extraction and use Eq. (4) with *x*, *b* and *W* as the qubits, local field and coupling strengths respectively to obtain samples from the quantum annealing. The frequency with which a qubit is observed to take value 1 is interpreted as the activation output $\sigma (Wx)$ in Eq. (4). Second, when $\sigma (W(x))$ is available, we use this as *x* in Eq. (5) along with $b^c$ and $W^c$ as input to the quantum annealer to provide the classification status. The final state of a qubit in any sample can be interpreted as an indication of whether the corresponding neuron in the model has fired.

### Feature extraction

Consider a network comprising two groups of nodes, *v* and *h*, with connections between each member of *v* and each member of *h* and no connections within *v* or *h*. The energy of this restricted Boltzmann machine model is

9 $$\begin{aligned} E(v,h) = -\sum _i b^v_i v_i - \sum _j b_j h_j - \sum _{i,j} v_i w_{ij} h_j \end{aligned}$$

and the joint probability is given by

10 $$\begin{aligned} p(v,h) = \frac{\exp (-E(v,h))}{\sum _v\sum _h \exp (-E(v,h))}. \end{aligned}$$

    

The resulting bipartite structure gives rise to analytic expressions for the conditional probabilities: the probability that *h* is on given *v* and the probability that *v* is on given *h*. Consequently, the conditional distribution *p*(*h*|*v*) is simple to compute, see for example^16^ for the derivation of the expression

11 $$\begin{aligned} p(h_j = 1 | v) = \sigma (b_j + (v^TW)_j) \end{aligned}$$

for $\sigma$ defined in Eq. (8).

As the first step in transfer of $f^1$ to the QPU, we assign *N* qubits as input nodes *v* and *M* qubits as output nodes *h*. For annealing, the known values of *v* will be realised by setting the strength of the local field biases, $b^v$, so that the *v* are effectively clamped on or off as appropriate. The local field biases of *h* are set to *b* and the coupling strengths between *v* and *h* are set to *W* with coefficients $w_{ij}$, from Eq. (4). Mapping these nodes ($v_i$, $h_j$) and coefficients ($b_i$, $c_j$, $w_{ij}$) to the QPU, and using quantum annealing to obtain samples, is equivalent to sampling a Bernouilli random variable from a suitably defined sigmoid distribution. In summary we use this equivalence to transfer weights from either a classically trained sigmoid activation layer within a neural network or a RBM to the appropriate number of qubits and associated parameter values. We then run quantum annealing and take samples. These samples correspond to low energy solutions.

As outlined above the classical samples come from Eq. (4). However the quantum samples arise from a probability distribution modified by a temperature coefficient to be estimated from the data^19^. We address this issue by introducing a parameter *S* and evaluate its sensitivity on the results. The purpose of this parameter is to align the classical and quantum Boltzmann distribution according to

12 $$\begin{aligned} f_1(v) = \sigma (S(Wv + b)). \end{aligned}$$

   

The classical neural network is then trained by using an adapted sigmoid layer with activation $\sigma (Sv)$ and adjusting the weights that are transferred to the QPU to *SW*.

### Classification

To use the QPU for classification, we transfer the classification map, $f^2$, to the QPU by assigning *M* qubits as feature nodes, *h*, and *K* qubits as output nodes $h^c$. In the case where the values of *h* are known, we set the local biases, $b^h$, so that *h* are clamped. The local biases of $h^c$ are set to $b^c$ and the coupling strengths between *h* and $h^c$ are set to $W^c$ with coefficients $w^c_{ij}$ from Eq. (6). In this network, the energy is

13 $$\begin{aligned} E(h,h^c) = -\sum _j b^h_j h_j - \sum _k b^c_k h^c_k - \sum _{j,k} h_j w^c_{jk} h^c_k. \end{aligned}$$

   

Quantum annealing will provide samples from $p(h,h^c)$. However, unlike the feature extraction setting, the on/off status of $h^c$ reflects the strength of $h_j w^c_{jk} + b^c_j$ on node $h^c_k$ and represents a count for each class. To encourage the entry of $h^c$ with the highest probability to be the only node on, we introduce a parameter $\lambda \ge 0$ and alter the energy function to

14 $$\begin{aligned} E(h,h^c) = -\sum _j b^h_j h_j - \sum _k b^c_k h^c_k - \sum _{j,k} h_j w^c_{jk} h^c_k + \lambda \sum _{k,l} h^c_k h^c_l. \end{aligned}$$

    

The effect of $\lambda$ will depend on the values of the other parameters. For a classification with 10 classes, as in the experiments below, we found that $\lambda =8$ where $k \ne l$ and $\lambda =0$ when $k = l$ worked well.

### Quadratic unconstrained binary model

We now explain how the quantum annealer will be used. As mentioned above, a quadratic binary model defines an energy-based network of binary random variables with real-valued parameters for biases and correlation weights. Energy minimisation involves finding the binary values of the model states that result in the lowest energy levels. This minimisation problem is also referred to as quadratic unconstrained binary optimisation, or QUBO.

#### Feature extraction

For feature extraction the energy of the network, connecting visible nodes *v* and feature or hidden nodes *h* with weights, *W*, biases, $b^v$ and *b*, respectively, as in Eq. (9), can be expressed using vector and matrix notation

15 $$\begin{aligned} E(v,h)=\,& {} [v h]^T Q [v h] \nonumber \\=\,& {} \left[ \begin{array}{cccccc} v_1&\ldots&v_N&h_1&\ldots&h_M \end{array} \right] \left[ \begin{array}{cccccc} b^v_1 &{} &{} &{} w_{11} &{} \ldots &{} w_{1M} \\ &{} \ddots &{} &{} \vdots &{} &{} \vdots \\ &{} &{} b^v_N &{} w_{1N} &{} \ldots &{} w_{NM} \\ &{} &{} &{} b_1 &{} &{} \\ &{} &{} &{} &{} \ddots &{} \\ &{} &{} &{} &{} &{} b_M \\ \end{array} \right] \left[ \begin{array}{l} v_1 \\ \vdots \\ v_N \\ h_1 \\ \vdots \\ h_M \end{array} \right] . \end{aligned}$$

#### Classification

For classification purposes the energy of the network, connecting feature nodes *h* and class nodes $h^c$ with weights, $W^c$, biases, $b^h$ and $b^c$, and parameter, $\lambda$, as in Eq. (14), can be expressed as

16 $$\begin{aligned} E(h,h^c)=\,& {} [h h^c]^T Q [h h^c] \nonumber \\=\,& {} \left[ \begin{array}{cccccc} h_1&\ldots&h_M&h^c_1&\ldots&h^c_K \end{array} \right] \left[ \begin{array}{cccccc} b^h_1 &{} &{} &{} w^c_{11} &{} \ldots &{} w^c_{1K} \\ &{} \ddots &{} &{} \vdots &{} &{} \vdots \\ &{} &{} b^h_M &{} w^c_{1M} &{} \ldots &{} w^c_{MK} \\ &{} &{} &{} b^c_1 &{} \lambda &{} \lambda \\ &{} &{} &{} &{} \ddots &{} \lambda \\ &{} &{} &{} &{} &{} b^c_K \\ \end{array} \right] \left[ \begin{array}{l} h_1 \\ \vdots \\ h_M \\ h^c_1 \\ \vdots \\ h^c_K \end{array} \right] . \end{aligned}$$

### Convolutional filters

Feature extraction using convolutional filtering is interesting for two reasons. First, it can lead to spatially invariant solutions. Second, by sharing a small number of weights, convolutional filters introduce sparsity into the connections between layers and nodes. To exploit the quantum annealing framework, we express the convolutional operation as *Wx*, where *W* is a sparse matrix of dimension $N \times FP$. Here *FP* is the resulting number of features after *P* small 2D filters have been passed over the image. The value of *FP* will depend on the size of the input image, including any padding, and the stride with which the filter passes over the input image. The matrix *W* is formed by tiling the filters, $\{W^1, W^2, \ldots , W^P\}$, in such a way that each member of each filter is coupled with the appropriate input nodes. This formation can be transferred to the QPU. This differs from the classical representation which uses tensor sum and multiplication to compute features.

How these weights form part of the energy system *Q* and replace W in Eq. (9) is illustrated below

17 $$\begin{aligned} \left[ \begin{array}{ccccccc} w^1_{1,1} &{} &{} &{} \dots &{} w^p_{1,1} &{} &{} \\ w^1_{1,2} &{} w^1_{1,1} &{} &{} \dots &{} w^p_{1,2} &{} w^p_{1,1} &{} \\ w^1_{1,3} &{} w^1_{1,2} &{} w^1_{1,1} &{} \dots &{} w^p_{1,3} &{} w^p_{1,2} &{} w^p_{1,1} \\ &{} w^1_{1,3} &{} w^1_{1,2} &{} \dots &{} &{} w^p_{1,3} &{} w^p_{1,2} \\ \dots &{} \dots &{} \dots &{} \dots &{} \dots &{} \dots &{} \dots \\ w^1_{2,1} &{} &{} &{} \dots &{} w^p_{2,1} &{} &{} \\ w^1_{2,2} &{} w^1_{2,1} &{} &{} \dots &{} w^p_{2,2} &{} w^p_{2,1} &{} \\ w^1_{2,3} &{} w^1_{2,2} &{} w^1_{2,1} &{} \dots &{} w_{2,3} &{} w^p_{2,2} &{} w^p_{2,1} \\ &{} w^1_{2,3} &{} w^1_{2,2} &{} \dots &{} &{} w^p_{2,3} &{} w^p_{2,2} \\ \dots &{} \dots &{} \dots &{} \dots &{} \dots &{} \dots &{} \dots \\ w^1_{3,1} &{} &{} &{} \dots &{} w^p_{3,1} &{} &{} \\ w^1_{3,2} &{} w^1_{3,1} &{} &{} \dots &{} w^p_{3,2} &{} w^p_{3,1} &{} \\ w^1_{3,3} &{} w^1_{3,2} &{} w^1_{3,1} &{} \dots &{} w^p_{3,3} &{} w^p_{3,2} &{} w^p_{3,1} \\ &{} w^1_{3,3} &{} w^1_{3,2} &{} \dots &{} &{} w^p_{3,3} &{} w^p_{3,2} \\ \dots &{} \dots &{} \dots &{} \dots &{} \dots &{} \dots &{} \dots \\ \end{array} \right] . \end{aligned}$$

   

A limiting factor as to the size of a neural net that can be transferred to the QPU is the number of nodes and couplers. Compared with a fully connected filter, a convolutional filter has fewer weights reducing the number of edges in the network and the coupling requirement. This increases the number of features that can be obtained from the QPU. For example, a convolutional layer input size $15 \times 15$ and 8 convolutional filters size $5 \times 5$ gives a *W* with dimensions $225 \times 288$ and can be transferred to the QPU. A fully connected layer with the same input size is limited to *W* with dimensions $225 \times 36$.

### Transfer to QPU

The weights and biases are physically transferred to the QPU in the sparse matrix format shown in Eqs. (15) and (16). Before the quantum annealing process, a mapping must be found that physically embeds the *Q* matrix on the D-Wave graph. This embedding is found using the available heuristic tool, minorminer^20^, at the beginning of the first run. The map is then reused in subsequent runs. The D-Wave sampler is called and the returned samples are ordered from lowest to highest energy and summed across classes to provide a consensus class score for classification.

## Experimental results

### Feature extraction on QPU

We investigate our hybrid classical quantum approach to feature extraction by transferring the computation of a convolutional layer from a classically trained NN on a CPU/GPU to the D-Wave GPU. A convolutional layer that passes *P* small $5 \times 5$ filters with stride 2 over *N* inputs creates *FP* features. To execute this layer a total of *N* + *FP* qubits and $5^2 FP$ couplers are required. Using D-Wave’s minorminer we found an embedding for $N=225$, $F=36$, $P=8$ creating a network with 513 qubits and 900 couplers. This was the largest suitable network we found and for comparison purposes defined the scope of the classical training. We trained a convolutional NN classifier with 60,000 centre cropped (from $28 \times 28$ to $15 \times 15$) and binarised training MNIST images. The network comprised a convolutional block (8 filters of dimension $5 \times 5$ which pass over the image with stride 2) followed by a sigmoid activation layer, a fully connected layer (connecting 288 features to 10 classes) and a softmax classification layer. The sigmoid activation layer (for forward and backward passes during training) was modified by a temperature coefficient S, $\sigma _S(x;S)=\sigma (Sx)$ and networks were trained for a range of values $S=1,2,3,4,5,6$ and 8. The inclusion of this parameter allows us to assess the sensitivity of the results to effective temperature which may misalign the classical and quantum Boltzmann distributions. A $Q_S$ matrix is set up for a given image *v* and each network. The local field biases, $b^v$, are set to -50 or 50 for $v_i=1$ and $v_i=0$ respectively. The feature biases, *b*, and coupling weights, *W*, are extracted from the trained convolutional block. These parameters form $Q_S$ as indicated in Eq. (17).

We compare the performance between quantum QPU and classical CPU/GPU computation in terms of feature extraction and subsequent classification on 100 unseen MNIST test digits using a classically trained neural network as defined in Eq. (6). The quantum results are obtained by framing feature extraction in quadratic form, Eq. (15), finding an embedding for Q, and using quantum annealing to return 500 samples from the probability distribution in Eq. (10). We use these samples to form our class predictions, as reported in Table 1. Accuracy is higher for the results based on the 100 samples with the lowest energy than on all the 500 samples. With the temperature coefficeint set to 4, the accuracy of the quantum approach closely matches the classical case ($86\%$ versus $87\%$).

**Table 1:** MNIST Digit Classification. QA prediction accuracy based on 100 samples with the lowest energy out of 500 samples *column 2*, all 500 samples *column 3* and classical accuracy *column 1* for 100 test images are shown for different temperature settings *rows*. The most consistent and highest level of accuracy with QA is seen with temperature coefficient 4.

Temperature coefficient	Accuracy (out of 100)		
	Classical	QA 100/500	QA 500/500
1	87	80	76
2	88	76	72
3	87	79	75
4	**87**	**86**	**81**
5	87	82	74
6	88	73	68
8	86	76	64

Most consistent values are in bold.

### Classification on QPU

We now investigate transferring the computation of $f^2$ from Eq. (5) to the QPU. An RBM classifier was trained following^21^ using MATLAB code available from the authors website (https://www.cs.toronto.edu/~hinton/MatlabForSciencePaper.html). The network consisted of an RBM layer for binary feature extraction and a fully connected classification layer. The RBM layer was pretrained in an unsupervised manner with MNIST data using the contrastive divergence algorithm. The classification layer was then added and the weights from the two layers fine-tuned using labelled MNIST data and the backpropagation algorithm^21^. In this way we leverage efficient classical algorithms and parallel computing. Once trained, we use the RBM layer to map from the image space ${\mathbb {R}}^N$ to binary feature space ${\mathbb {B}}^M$. For our experiments, we choose $\{N,M\}$ to be $\{225,145\}$, $\{361,230\}$ and $\{729,465\}$ with the 60,000 MNIST training images centre cropped from $28 \times 28$ to $15 \times 15$, $19 \times 19$ and $27 \times 27$ respectively.

By assigning qubits to each of the binary feature nodes, *h*, and each class node, $h_c$, of the RBM classifier network we can embed the network directly on to the QPU and use quantum annealing to sample from the network. In this context, we clamp the known binary feature values, using strong bias values, $b^h$, to force the units to be on or off, and determine from the samples the state of the class nodes. These values can then be used to determine the most likely class for a test image. To constrain the most likely class to one value we set $\lambda =8$ in Eq. (14). The matrix *Q* is built as in Eq. (16) using $b^h$ and the parameters extracted from the RBMs, $b^c$ and $W^c$. Before quantum annealing can proceed, a mapping is found that physically embeds the *Q* matrix on the D-Wave graph. This embedding is found using minorminer^20^.

For each model, 10 samples and 100 samples are obtained from the quantum annealer for 100 test images. These sets of samples are used to determine the most likely image class. Table 2 shows the number of correctly classified test images when 10 or 100 samples were obtained from quantum annealing and compares this result with the classical case. We see that the quantum approach gives comparable performance to the classical version, and for the largest scale computation is able to match the $99\%$ accuracy.

**Table 2:** MNIST Classification using a hybrid quantum classical RBM with three feature sizes (145, 230 and 465).

Accuracy (out of 100 Images)			
Number of samples per image	10	100	1
Model	QA10	QA100	classical
145	81	87	97
230	83	94	98
465	97	99	99

**Table 3:** Comparison between QPU and GPU execution times in microseconds.

Model input size	145	230	465
QPU anneal time per sample	20.0	20.0	20.0
QPU readout time per sample	140.8	160.9	189.6
QPU delay time per sample	20.5	20.5	20.5
Total QPU sampling time for one sample	181.3	201.4	230.1
GPU 1 image - time per image	1215.6	1050.8	1260.7
GPU 10 images - time per image	272.5	251.8	231.7
GPU 100 images - time per image	38.5	37.6	18.7
GPU 1000 images - time per image	3.0	3.0	3.0

### Timings

The D-Wave system handles one QUBO problem at a time and in our context the combined size of images and networks is limited by the number of qubits and couplers. There are also associated pre-processing, overhead and post-processing timing costs. As such D-Wave is not currently an efficient way to process state-of-the-art neural networks. Also, discovering a good QUBO solution may require more than one sample, proportionally increasing the total QPU time. In our timing experiments we used 10, 100 and 1000 samples. For comparison between QPU and GPU in Table 3 we report the total QPU sampling time for one sample, under different model input sizes (145, 230 and 465), as $181.3 \mu s$, $201.4 \mu s$ and $230.1 \mu s$ respectively. The total QPU sampling time comprises an anneal time ($20 \mu s$), a delay time ($20.5 \mu s$) and a readout time which increases with input size ($140.8 \mu s$, $160.9 \mu s$ and $189.6 \mu s$). Based on computations on a single TITAN Xp GPU we estimate the equivalent GPU time to process one image (input size 145, 230 and 465) to be $1215.6 \mu s$, $1050.8 \mu s$ and $1260.7 \mu s$ respectively. In this respect, the QPU timings look promising, and we also note that the anneal time is insensitive to image size. However processing just one image is not an efficient way to use a GPU, as seen by the time reduction per image for input size 465 when 10 ($231.7 \mu s$), 100 ($18.7 \mu s$) and 1000 ($3.0 \mu s$) images are processed. The results suggest that in order to realise a quantum speedup of at least one order of magnitude, at least 10 images need to be processed in parallel. This may be achieved in the near future with novel methods to make better use of available qubits^22^ and with expected increases in the number of qubits and couplers on quantum machines.

## Discussion and conclusions

Quantum annealing, which allows us to tackle problems in QUBO form, has been successfully exploited to solve several graph-based discrete optimisation problems^23,24^. In other related work, reviewed by^10^, quantum annealers have been adapted for machine learning classification, and in particular for training hybrid models including RBMs. Caldeira et al.^18^ show that RBMs implemented on D-Wave hardware perform well, show some classification performance on small datasets but do not offer a broadly strategic advantage for this task. In order to tackle real-world datasets, within the D-Wave constraints, it is necessary to make approximations^11^ that cause loss of expressive power and consequently do not outperform classical approaches. Nath et al.^10^ find that several challenges need to be overcome before quantum annealing can be widely used as a complete replacement for classical computation in this context. This includes increasing the number of qubits and the connections between them.

In this work we have focused instead on a specific aspect of the deep learning pipeline, showing that it is possible to frame a classification task in QUBO form using the weights from a classically trained neural network. This problem can then be sent to D-Wave’s QPU for one-step quantum annealing. In summary, given a trained network, D-Wave can be used to evaluate it. This strategy may be viewed as an example of a hybrid quantum-classical algorithm^25^. Our computational results indicate that this new approach can produce comparable accuracy to the purely classical case, while offering potential for computational speed-up.

Regarding timings, we have focused on the annealing time (around 20 $\mu s$). Currently, there are time overheads (access, programming, sampling and post-processing) involved. These could be addressed by engineering advancements that improve streaming to and from the QPU for specific tasks such as classification.

We addressed two barriers to scaling up: binary input states which restrict the types of data that can be analysed and the number of couplings between nodes limited by the physical D-Wave graph; and the required number of model variables. We showed that real valued features can be considered as biases that can be added directly to the biases of connecting nodes or subject to pooling and connecting node constraints. This illustration opens up the possibility of handling different types of input features including those derived from other systems or multi-faceted features from complex systems. We introduced convolutional couplings which serve two main purposes. First, down-sampling the input to a) reduce the size of the network and hence the number of parameters needed (especially important for quantum computers), (b) reduce the computational cost, and (c) improve generalisation. Second, adding non-linearity, required for better model expressiveness, to the linear maps. Convolutional filters also reduce the connectivity load, and by extracting features from spatial settings they can improve model expressiveness.

In summary, providing neural networks with a quantum engine has the potential, assuming the pipeline for streaming data to the quantum computer can be made more efficient, to obtain classification results from high dimensional sources with speeds at least an order of magnitude greater than classical analogs.

## Data Availability

The data from this study is available at http://dx.doi.org/10.5525/gla.researchdata.1409.
